# GVBD rate is an independent predictor for pregnancy in ICSI patients with surplus immature oocytes

**DOI:** 10.3389/fendo.2022.1022044

**Published:** 2023-01-09

**Authors:** Meng Wang, Qiyu Yang, Jing Liu, Juan Hu, Dan Li, Xinling Ren, Qingsong Xi, Lixia Zhu, Lei Jin

**Affiliations:** ^1^ Reproductive Medicine Center, Tongji Hospital, Tongji Medical College, Huazhong University of Science and Technology, Wuhan, China; ^2^ Department of Oncology, Tongji Hospital, Tongji Medical College, Huazhong University of Science and Technology, Wuhan, China

**Keywords:** pregnancy prediction, germinal vesicle breakdown, logistic regression analysis, decision curve analysis, *in vitro* maturation

## Abstract

**Introduction:**

It was reported that there were still up to 30% immature retrieved oocyte at germinal vesicle (GV) or metaphase I (MI) stage. Whether the spontaneous maturity competency of immature oocytes associated to the clinical outcome of *in vitro* fertilization (IVF) cycles remains unclear and unexplored. This study aimed to investigate how the oocyte developmental parameters in *in vitro* maturation (IVM) affect clinical outcomes of intracytoplasmic sperm injection (ICSI) cycles.

**Methods:**

This retrospective cohort study included couples undergoing ICSI in a university-affiliated hospital. Surplus immature oocytes during ICSI were collected and cultured *in vitro*. The numbers of germinal vesicle (GV) oocytes undergoing GV breakdown (GVBD) and polar body 1 extrusion within 24 h culture were recorded. The main outcome measurements were demographic baselines and oocyte developmental parameters in IVM associated with pregnancy outcomes.

**Results:**

A total of 191 couples were included with an overall GVBD rate of 63.7% (327/513) and oocyte maturation rate of 46.8% (240/513). 53.4% (102/191) of them had embryos transferred freshly, which originated from metaphase II oocytes that matured spontaneously *in vivo*, and 60.8% (62/102) got pregnant. Among factors with a *P*-value < 0.2 in univariate logistic regression analyses of pregnancy correlation, GVBD rate (OR 3.220, 95% CI 1.060-9.782, *P*=0.039) and progesterone level on human chorionic gonadotropin (HCG) day (OR 0.231, 95% CI 0.056-0.949, *P*=0.042) remained significant in the multivariate model. The area under the curve (AUC) of the predictive nomogram was 0.729 (95% CI 0.632-0.826) with an acceptable calibration. Moreover, decision curve analyses illustrated the superior overall net benefit of models that included the GVBD rate in clinical decisions within a wide range of threshold probabilities.

**Conclusion:**

In conclusion, GVBD rate and progesterone level on HCG day may be associated with pregnancy outcomes in infertile couples during the regular ICSI procedure. An elevated GVBD rate within 24 h may greatly increase the likelihood of pregnancy in infertile couples during ICSI. This preliminary study may optimize clinical pregnancy prediction, which provides support in decision-making in clinical practice.

## Introduction

Assisted reproductive technology (ART) is an optimal method for infertility treatment (1). Although ART procedures are becoming a standardized process, a prediction of pregnancy chances in an ART cycle help clinicians to formulate individualized treatment strategy (2). An effective model should cover all possible relevant predictive factors. Unfortunately, although various predictive models have been established for pregnancy outcomes after *in vitro* fertilization (IVF) (3–5), the putative predictors identified in these models were diverse from each other, and currently, there is no consensus on which factor was the golden predictor of IVF success and the base of decision making (6). A systematic review analyzed nine factors associated with pregnancy outcomes after IVF, and it was demonstrated that some baseline characteristics including age, subfertility duration, and parameters of ovarian function may be predictors of pregnancy (6). Expect these frequently used factors, whether there are some other underappreciated but effective predictors for IVF success.

Oocyte maturation *in vivo* was regarded as a result of natural selection. During the process of controlled ovarian hyperstimulation (COH), the continuing injection of exogenous gonadotropin induced follicular growth and maturation to obtain a considerable number of available oocytes, which was able to guarantee the probability of clinical pregnancy for success (7, 8). However, it was reported that there were still up to 30% of immature retrieved oocytes at the germinal vesicle (GV) or metaphase I (MI) stage (9). Commonly, the immature oocytes were abandoned, while they may be matured *in vitro* for clinical use under certain circumstances, namely rescue *in vitro* maturation (R-IVM), although the developmental competency of these oocytes was usually lower than that of their sibling matured oocytes *in vivo*. Moreover, the reason why some of these immature oocytes may resume meiosis to the metaphase II (M II) stage during *in vitro* maturation (IVM) and the others may not be able to accomplish the progress to maturity (10, 11) is underlying. Whether the spontaneous maturity competency of immature oocytes is associated with the clinical outcome of IVF cycles remains unclear and unexplored.

As no studies have ever explored the correlation between developmental competence of R-IVM procedures and the chance of IVF success, the goal of the current study was to investigate how the oocyte developmental parameters in IVM affect clinical outcomes of regular IVF cycles and to explore which factors can predict clinical outcomes independently.

## Materials and methods

### Study design and study population

This was a retrospective cohort study conducted at Reproductive Medical Center, Tongji Hospital, Tongji Medical College, Huazhong University of Science and Technology. The Institutional Review Board of Tongji Hospital approved this study (No: TJ-IRB20201209), and each participant gave written informed consent. The study population included infertility couples with surplus immature oocytes for IVM during regular intracytoplasmic sperm injection (ICSI) treatments between January 2018 and December 2019 in Tongji Hospital. The exclusion criteria were as follows : (1) no available GV oocytes ; (2) preimplantation genetic testing (PGT) cycles; (3) couples with identified gene mutation correlated to fertility; (4) total fertilization failure cycles; (5) frozen or donated oocyte cycles; (6) surgical sperm retrieval including testicular sperm aspiration (TESA), percutaneous epididymal sperm aspiration (PESA), and microsurgical epididymal sperm aspiration (MESA).

### Oocytes retrieval procedure

COH was conducted as previously described (12). The included participants experienced either Gonadotropin-releasing hormone (GnRH) agonist or GnRH antagonist protocol. The dosage and duration of gonadotrophin administrated were based on ovarian response. Recombinant human chorionic gonadotropin (HCG) administration in a dosage of 10 000 IU was used for trigger when 2-3 larger follicles over 18 mm in diameter were detected. Oocyte retrieval was guided by transvaginal ultrasound approximately 36-38 hours later after trigger and all follicles with a diameter of no less than 12 mm were aspirated. Cumulus-oocyte complexes (COCs) were collected and incubated in an IVF medium (Vitrolife, Sweden) at 37 ℃ with 6% CO_2._ Degranulation of COCs was processed after 2-3 h culture. Cumulus cells were removed mechanically after treatment in a medium supplemented with 80 IU hyaluronidase (Vitrolife, Sweden). Subsequently, the oocytes were gathered and the maturity was evaluated.

### Embryo culture and morphological evaluation

Mature oocytes at the MII stage were fertilized by ICSI and cultured in a G1-plus medium (Vitrolife, Sweden). The presence of two pronuclei (2PN) 16-18 h after insemination was considered normal fertilization. Then, the zygotes were continuously cultured to the cleavage stage until day 3 for morphological evaluation, and one or two high-quality embryos scored as Grade 1 and Grade 2 were transferred. The main parameters of morphological evaluation were the number of blastomeres, the percentage of fragmentation, and the variation in blastomere symmetry. High-quality embryos were referred to normally fertilized embryos with more than 6 uniform blastomeres and <20% fragmentation on day 3. Grade 1 embryos were defined as normally fertilized embryos with 7-10 equal blastomeres and <10% fragmentation on day 3. Grade 2 embryos were high-quality embryos not fulfilling the Grade 1 criteria.

### Clinical outcome measurement

For patients with D3 embryo transfer freshly, persistent luteal supports were administrated. Serum HCG levels were measured 14 days after embryo transfer, and a positive result referred to biochemical pregnancy. Clinical pregnancy was defined as the ultrasound visualization of intrauterine gestational sacs with an active fetal heart approximately five weeks after embryo transfer.

### IVM procedure

Immature oocytes at the GV stage after degranulation were cultured in a G1-plus medium (Vitrolife, Sweden) in an incubator with 6% CO_2_ and 5% O_2_ at 37 ℃. The parameters of oocyte nuclear maturation were mainly germinal vesical breakdown (GVBD) and polar body 1 (PB1) extrusion. The numbers of GV oocytes undergoing GVBD and PB1 extrusion within 24 h culture were recorded, which were the numerators of GVBD rate and oocyte maturation rate in IVM, respectively. The corresponding denominators were both the number of GV oocytes collected.

### Statistical analysis

For continuous variables, the distribution was tested using the Shapiro-Wilk normality test. The normally distributed continuous variables were presented as mean ± standard deviation, and the non-normally distributed variables were presented as median (first quartile (Q1), third quartile (Q3)). For categorical variables, the data were presented as % (n). Baseline characteristics were compared between non-pregnancy and pregnancy groups using Student’s *t* test, non-parametric rank-sum Mann-Whitney *U* test, Chi-square test, and Fisher’s Exact test according to type and distribution of data as appropriate. Logistic regression analyses were performed to assess potential predictors associated with pregnancy outcomes. The variables with *P* values less than 0.2 in univariate analyses were included in the final model for multivariate logistic regression analyses. Collinearity diagnostics were performed, and variables with good collinearity with other factors in the model were excluded. Nomogram with distribution was established using R software. The discrimination of the nomogram was evaluated using the concordance index (C-index) and the area under curve (AUC) of the receiver operating characteristic curve (ROC), and the calibration was measured using the calibration curve and the Hosmer-Lemeshow calibration test (13). Decision curve analyses were performed to evaluate the clinical utility of different predictive models (14). The data analyses were performed using SPSS (version 26.0, IBM, USA), R (version 4.0.4), and the R packages as follows: rms (version 6.2-0), regplot (version 1.1), pROC (version 1.18.0), and rmda (version 1.6). A two-tailed *P* value <0.05 was considered to be of statistical significance.

## Result

### Demographic and clinical characteristics of participants

A total of 191 couples with surplus GV oocytes obtained during regular ICSI treatments between January 2018 and December 2019 were enrolled in the study. In the 191 couples, 513 immature oocytes at the GV stage were collected and cultured, and 327 of them underwent spontaneous GVBD within 24 h with an overall GVBD rate of 63.7% (327/513). In addition, a total of 240 oocytes extruded the first polar body within 24 h *in vitro* with an overall oocyte maturation rate of 46.8% (240/513). The median (Q1, Q3) of GV oocytes, GVBD rate, and oocyte maturation rate were 2 (1, 4), 75% (33%, 100%), and 50% (0%, 100%) respectively. In all 191 cycles, M II oocytes resulting from IVM can be obtained in 135 cycles.

Of the enrolled 191 couples, 53.4% (102/191) of the cycles had embryos transferred in fresh on day 3, and 60.8% (62/102) got pregnant. The baseline characteristics of the participants in fresh embryo transfer cycles were presented in **Table 1**. The patients who were confirmed as clinical pregnancy have more antral follicle count (AFC, *P*=0.034), higher basal anti-müllerian hormone (AMH, *P*=0.049) level, shorter infertility duration (*P*=0.036), and fewer overall ART attempts (*P*=0.004). There were no significant differences between the pregnancy group and the non-pregnancy group regarding other characteristics.

**Table 1:** Demographics and clinical characteristics of participants.

Factors	Non-pregnancy (n=43)	Pregnancy (n=59)	*P* value
Age, yr	31.1 ± 4.4	29.9 ± 3.3	0.153
BMI, kg/m^2^	21.3 (19.5, 23.9)	21.2 (19.5, 22.9)	0.647
FSH, mIU/mL	7.4 (6.5, 8.5)	7.3 (6.2, 8.5)	0.655
AFC	12 (9, 18)	16 (11, 22)	0.034
AMH, ng/mL	3.8 (2.8, 6.1)	5.9 (3.3, 8.7)	0.049
Infertility type			0.828
Primary, %	81.4 (35/43)	79.7 (47/59)	
Secondary, %	18.6 (8/43)	20.3 (12/59)	
Infertility duration, yr	4.0 (2.5, 5.0)	3.0 (2.0, 4.0)	0.036
Infertility cause			0.680
Female factor, %	46.5 (20/43)	33.9 (20/59)	
Male factor, %	27.9 (12/43)	35.9 (21/59)	
Mixed factors, %	23.3 (10/43)	27.1 (16/59)	
Unexplained, %	2.3 (1/43)	3.4 (2/59)	
Overall ART attempts	2 (1, 2)	1 (1, 1)	0.004
COH protocol			0.251
GnRH-agonist, %	69.8 (30/43)	79.7 (47/59)	
GnRH-antagonist, %	30.2 (13/43)	20.3 (12/59)	
Gonadotrophin duration, d	10 (9, 11)	10 (9, 11)	0.263
Gonadotrophin dosage, IU	2400 (1973, 3000)	2145 (1575, 2850)	0.203
No. of large follicle	10.9 ± 4.4	11.6 ± 4.5	0.460
Estradiol level on HCG day, pg/mL	2097 (1626, 2987)	2074 (1506, 2729)	0.773
Progesterone level on HCG day, ng/mL	0.7 (0.5, 0.9)	0.6 (0.4, 0.9)	0.071
Endometrium thickness on HCG day, mm	11.7 (9.6, 14.0)	12.3 (10.3, 14.5)	0.319
GVBD rate, %	100 (0, 100)	100 (50, 100)	0.156
Oocyte maturation rate in IVM, %	50 (0, 100)	50 (20, 100)	0.424
No. of oocyte retrieval	13.1 ± 5.5	13.5 ± 5.1	0.672
Matured oocytes rate, %	69.3 ± 0.2	68.8 ± 0.2	0.871
Fertilization rate, %	67 (60, 86)	75 (60, 88)	0.357
No. of embryo transfer			0.227
1	83.7 (36/43)	91.5 (54/59)	
2	16.3 (7/43)	8.5 (5/59)	
Grade of embryo transferred			0.599
Grade 1, %	62.8 (27/43)	67.8 (40/59)	
Grade 2, %	37.2 (16/43)	32.2 (19/59)	

The normally distributed continuous variables were presented as mean ± standard deviation, and the non-normally distributed variables were presented as median (first quartile, third quartile). For categorical variables, the data were presented as % (n). BMI, body mass index; FSH, follicle stimulating hormone; AFC, antral follicle count; AMH, Anti-müllerian hormone; ART, assisted reproductive technology; IVF, in vitro fertilization; ICSI, intracytoplasmic sperm injection; COH, controlled ovarian hyperstimulation; GnRH, gonadotropin-releasing hormone; HCG, human chorionic gonadotropin; GVBD, germinal vesicle breakdown; IVM, in vitro maturation.

### Logistic regression analyses for pregnancy outcomes prediction

Univariate logistic regression analyses were performed to explore the potential factors associated with pregnancy outcomes. The factors with a *P*-value < 0.2 were as follows: age, AFC, AMH, infertility duration, overall ART attempts, progesterone level on HCG day, and GVBD rate (**Table 2**). All the above-mentioned variables showed no significant collinearity and were then included in the multivariate model. As **Table 2** shown, the GVBD rate greatly increased the likelihood of pregnancy (odds ratio, OR 3.220, 95% confidence interval, 95% CI 1.060-9.782, *P*=0.039), while conversely, an increase in progesterone level on HCG day decreased the pregnancy odds (OR 0.231, 95% CI 0.056-0.949, *P*=0.042). The other included variables showed no statistical significance when predicting pregnancy in the multivariate regression analysis model (*P* > 0.05).

**Table 2:** Univariate and multivariate logistic regression.

Variables	Univariate		Multivariate	
	OR (95% CI)	*P* value	OR (95% CI)	*P* value
Age	0.922 (0.830-1.025)	0.134	1.004 (0.878-1.147)	0.956
AFC	1.084 (1.010-1.162)	0.025	1.078 (0.974-1.194)	0.146
AMH	1.109 (0.988-1.243)	0.078	1.029 (0.874-1.211)	0.732
Infertility duration	0.882 (0.742-1.048)	0.152	0.895 (0.728-1.100)	0.291
Overall ART attempts	0.647 (0.379-1.104)	0.110	0.698 (0.398-1.223)	0.209
Progesterone on HCG day	0.299 (0.087-1.022)	0.054	0.231 (0.056-0.949)	0.042
GVBD rate	2.802 (1.009-7.783)	0.048	3.220 (1.060-9.782)	0.039

OR, odds ratio; CI, confidence interval; AFC, antral follicle count; AMH, Anti-müllerian hormone; ART, assisted reproductive technology; HCG, human chorionic gonadotropin; GVBD, germinal vesicle breakdown.

### Construction and validation of the nomogram

**Figure 1:**
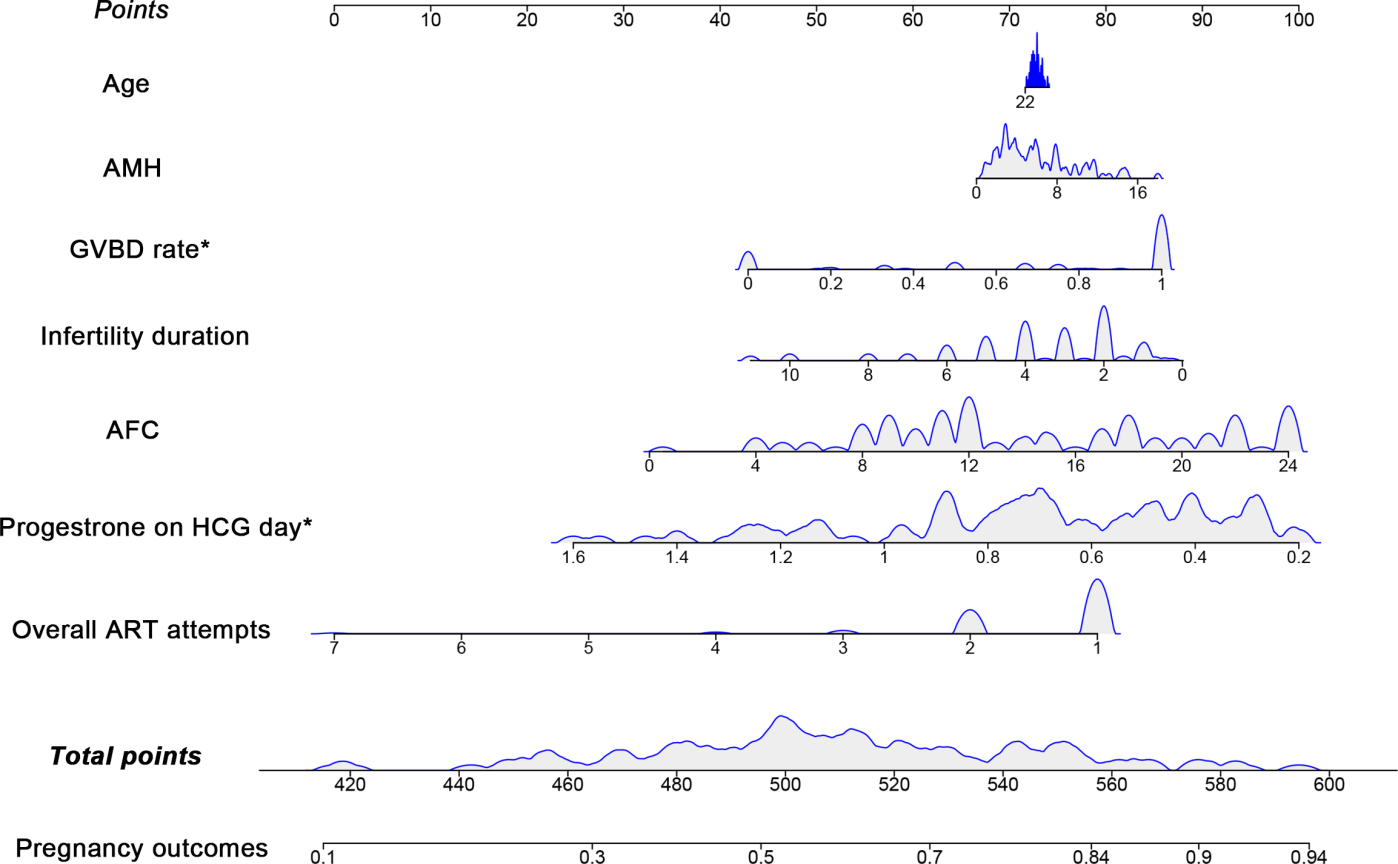
Nomogram with covariate distribution for predicting pregnancy using the multivariate logistic regression. The variables included in the nomogram were age, AMH, GVBD rate, infertility duration, AFC, progesterone on HCG day and overall ART attempts. Corresponding points were computed by drawing vertical lines from each variable axis upward to the point axis in the first row. The total points were then summed up and marked downward to the probability of the pregnancy outcomes in the last row. The variables marked with “*” were factors associated with pregnancy outcomes in the nomogram.

**Figure 2:**
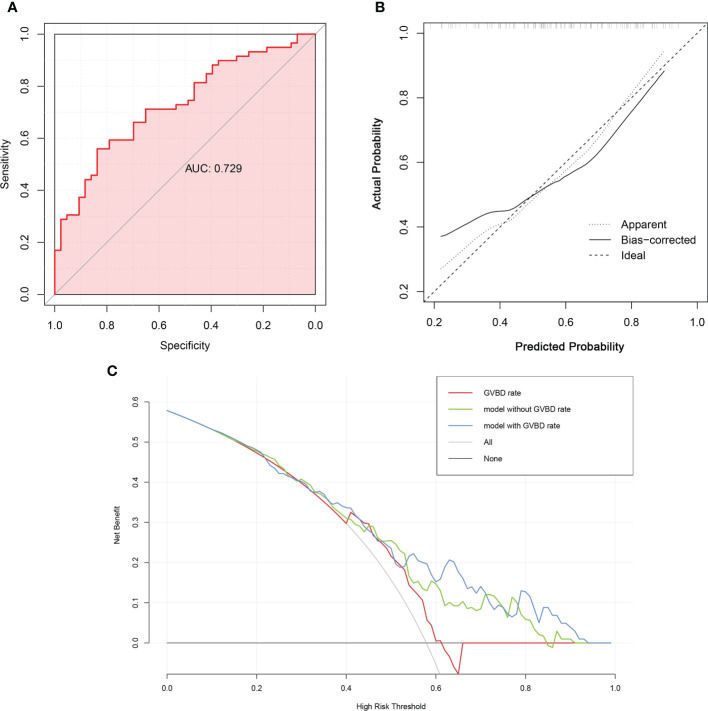
The validation of the multivariate logistic regression model. **(A)**. The discrimination was evaluated using the area under curve (AUC) of the receiver operating characteristic curve (ROC). **(B)**. The calibration was measured using calibration curve. The *x*-axis was the predicted probability of pregnancy, and the *y*-axis indicated the actual observed probability of pregnancy. **(C)**. Decision curve analyses of logistic regression models. The net benefit over a spectrum of probability thresholds was calculated to illustrate the accuracy of three models using decision curve analyses. The red curve represented a logistic regression model using GVBD rate only. The green line assumes the nomogram without GVBD rate, whereas the blue one plotted the nomogram with GVBD rate.

The predictive nomogram with covariate distribution was constructed using the seven variables included in the multivariate logistic regression model (**Figure 1**). The validation of the nomogram showed that the model had a c-index of 0.729 and a moderate AUC of 0.729 (95% CI 0.632-0.826) to discriminate patients who got pregnant from those who failed to be pregnant, with a Youden’s index of 0.396 (**Figure 2A**). Moreover, the Hosmer-Lemeshow goodness-of-fit test had a chi-square value of 4.728 (df=8) with a non-significant *P*-value of 0.786, and as **Figure 2B** exhibited, the calibration plot showed acceptable consistency between the bias-corrected curve and the ideal reference line with a mean absolute error of 0.048, indicating that the model was well-fitted with satisfactory calibrate ability. Furthermore, the decision curve analyses illustrated that the nomogram with a GVBD rate had a superior overall net benefit for clinical decisions within a wide range of threshold probabilities (range from 0.4 to 1.0) compared to the nomogram without a GVBD rate and the GVBD rate only **(** **Figure 2C**), particularly at higher threshold probabilities.

## Discussion

In the current retrospective cohort study, we established a multivariate logistic regression model with satisfactory discrimination and calibration to explore the potential predictors of pregnancy in patients with surplus immature oocytes for R-IVM during the regular ICSI procedure, and we found that the GVBD rate together with progesterone level on HCG day may be two independent factors associated with the likelihood of pregnancy. An increased GVBD rate within 24 h may improve the pregnancy odds and have some clinical utility in infertile couples appealing to ICSI treatment regarding pregnancy outcomes prediction.

Data regarding potential predictors of pregnancy in IVF treatments were numerous (6), while the exploration of the association between clinical characteristics and pregnancy chances was still a hotspot in the research field of reproductive medicine. Serum AMH level (15), female age (16), morphokinetics of embryo development (17), sperm DNA integrity (18), endometrial thickness and progesterone level on HCG day (19, 20), and many other factors have been proved to be candidate predictors of pregnancy outcome in IVF cycles. However, the predictive accuracy of these factors was various and the predictive ability was even conflicting sometimes (15). Moreover, there was a lack of a reliable and widely accepted predictor among these candidates, and no consensus was reached on which one was the most clinically relevant (6). In this study, univariate logistic regression analyses were performed and seven potential factors associated with clinical pregnancy, including age, AMH, AFC, overall ART attempts, infertility duration, progesterone on HCG day, and GVBD rate, were included in a well-fitted multivariate model. It was found that the GVBD rate may increase the likelihood of pregnancy, while the increase of progesterone level on HCG day may decrease the pregnancy odds. Our findings confirmed that GVBD may act as a novel reliable independent factor that could help in predicting pregnancy among the population of infertile couples during ICSI treatments.

The biological explanations for GVBD rate as a positive predictor of pregnancy chance most likely lay in the following aspects. First, the mature promoting factor (MPF), a complex consisting of cyclin-dependent kinases 1 (CDK1) and cyclin B, was essential for oocyte maturation and growth (21). It was well-known that the resumption of oocyte meiosis was induced by the activation of MPF (22), and the functional deficiency of MPF resulted in GV arrest (23). The presence of GVBD and the extrusion of the first polar body during the IVM procedure indicated the normal regulating function of MPF in oocyte maturation, which was further associated with the process of oocyte growth and fertilization. Thus, the GVBD rate might act as an indirect indicator of subsequent embryonic outcomes. Another possible mechanism was that the mature oocytes for fertilization shared a common maternal follicular environment with sibling immature oocytes for IVM. It has been demonstrated that crosstalk between oocytes and the microenvironment impacted oocyte quality and function (24). Moreover, metabolic components (25), energy consumption (26), and oxidative stress (27) in the surroundings were also associated with oocyte growth and quality. In addition, the epigenetic events in the polar body might also regulate the maturation and development competency of sibling oocytes (28). Therefore, the development potential of oocytes and subsequent embryos can be influenced by their surroundings, and the developmental parameters of the sibling immature oocytes at GV and MI stages, such as GVBD rate, might be able to predict pregnancy chances.

Among the included participant, 18 (9.4%) cycles were with a proportion of immature GV oocytes of more than 50%. The numbers of fertilized oocytes and available embryos of these specific patients were also limited. Although some of them had experienced embryo transfer freshly, the chance of a successful pregnancy was still slight. We speculated that a potential disordered regulating network may exist in the growth and development of the oocytes in such patients. It has been reported that mutation of several genes, such as TUBB8, PATL2, can lead to oocyte maturation and development disorder (29, 30). Although whole exome sequencing (WES) had been performed to exclude the possibility of relevant gene mutation, the process of oocyte development is complicated and precise, there may be aberrant regulation in other components, including RNA and protein, interfering with the maturation of oocytes, impairing oocyte developmental potentiality, and finally influencing pregnancy outcomes.

Human immature oocytes collected from the regular IVF process have offered embryologists the possibility to optimize ART procedure. IVM technique, as a supplement and modification of regular COH and IVF treatments, can increase the number of available embryos, particularly in patients who had poor ovarian reserve or oocyte maturation disorders (31). Moreover, it was becoming a routine method applied to fertility preservation among patients who were at risk of ovarian function loss due to various reasons (32). In addition, immature oocytes can be utilized for sperm selection. The spermatozoa which can bind to zonal pellucida (ZP) of immature oocytes may have better fertilization potential compared to those that failed to bind (33). A selection of these ZP-bound spermatozoa for ICSI can significantly improve fertilization rate and embryo developmental competency (34). Furthermore, oocytes resulting from IVM can help to identify the causes of couples with total fertilization failure as previously reported (35, 36). In our study, we found that the GVBD rate was an independent factor for clinical pregnancy prediction in infertile couples attending ICSI treatment, providing a new insight into the application of immature oocytes in clinical utility rather than being abandoned.

Another factor found to be detrimentally associated with pregnancy outcomes in the current study was the increased progesterone level on HCG day. Various studies have indicated that elevated serum progesterone levels have adverse impacts on IVF outcomes, mainly by affecting oocyte and embryo quality and endometrial receptivity (37, 38). In our previous study, we found that among the patients who underwent fresh embryo transfer with serum progesterone levels on HCG day no more than 1.5 ng/mL, there was still a negative correlation between progesterone level and pregnancy outcomes, indicating a detrimental effect of a high level of progesterone on oocyte competence (38). Moreover, progesterone levels might also impact the fate of immature oocytes. In rhesus monkeys, it was found that intrafollicular progesterone might regulate oocyte maturation (39). Similarly, another study demonstrated that progesterone concentration in follicular fluid was negatively linked to the developmental competence of immature bovine oocytes (40), revealing a possible impact of progesterone level on immature oocytes in mammals. Elevated progesterone levels influenced the growing environment of follicles, thus regardless of the maturity, the developmental competencies of oocytes were equally affected, which indirectly confirmed the consistency of the developmental competencies of matured oocytes and immature oocytes in the same pool. Therefore, the GVBD rate in the study can be used as a candidate for pregnancy prediction. However, the relationship between developmental parameters of immature oocytes and progesterone level on HCG was still ambiguous, and more studies needed to be conducted to explore it further.

In the current study, we have performed several tests to validate the discrimination and calibration of the model. It has been shown that the predictive logistic regression model was well-fitted with a satisfactory discriminate and calibrate ability, indicating the accuracy and effectiveness of the current model in predicting pregnancy outcomes. In recent years, there were a growing number of studies focusing on pregnancy prediction using artificial intelligence (41, 42). Zhu et al. have established a model for clinical pregnancy outcomes, and they found that mitochondrial DNA copy numbers may help in the prediction of pregnancy (43). Similarly, Zhang et al. also reported that a supporting vector machine model with basic characteristics can also achieve an AUC of 0.70 in cumulative pregnancy rate prediction (44). The AUCs of artificial intelligence models for pregnancy prediction which integrated widely accepted factors mainly ranged from 0.7 to 0.9, thus it is compressive to find other weighting factors to improve the efficiency of pregnancy prediction. Our results provided a new weight index to help in optimizing the pregnancy outcome prediction in IVF treatments.

A major strength of this study was that it provided a novel predictor of pregnancy outcomes in infertile couples attending ICSI treatments, which offers immature oocytes a new application in clinical utility and helps in optimizing the pregnancy outcome prediction. However, there were still several limitations. At first, it was a retrospective study with limited sample size. A multicenter study with a larger sample size is needed. Moreover, the results and conclusions of the current study were preliminary. Mechanism studies were urgent to explore the biological relationship between GVBD rate and pregnancy chances in ICSI treatments. Furthermore, the subjects included in our study were those couples who obtained surplus immature oocytes during regular ICSI treatments, and there may be a selection bias in the population investigated, thus our findings lack universality among the population of infertile patients.

In conclusion, GVBD rate and progesterone level on HCG day may be associated with pregnancy outcomes in infertile couples during the regular ICSI procedure. An elevated GVBD rate within 24h greatly increased the likelihood of pregnancy, and it optimizes clinical pregnancy prediction, which may help in decision-making in clinical practice.

## Data availability statement

The original contributions presented in the study are included in the article/supplementary material. Further inquiries can be directed to the corresponding author.

## Ethics statement

This study was approved by the Ethical Committee of Tongji Hospital (No: TJ-IRB20201209). The patients/participants provided their written informed consent to participate in this study.

## Author contributions

QX, LZ, and LJ conceived the study and have full access to the raw data; MW and LZ wrote the paper; JL, JH, DL, and XR collected the data; MW and QY analyzed the data. All authors contributed to the article and approved the submitted version.

## References

[1] De Geyter C. Assisted reproductive technology: Impact on society and need for surveillance. Best Pract Res Clin Endocrinol Metab (2019) 33:3–8. doi: 10.1016/j.beem.2019.01.004 30799230

[2] Sigman M. Introduction: Personalized medicine: what is it and what are the challenges? Fertil Steril (2018) 109:944–5. doi: 10.1016/j.fertnstert.2018.04.027 29935651

[3] Vaegter K Lakic T Olovsson M Berglund L Brodin T Holte J. Which factors are most predictive for live birth after in vitro fertilization and intracytoplasmic sperm injection (IVF/ICSI) treatments? analysis of 100 prospectively recorded variables in 8,400 IVF/ICSI single-embryo transfers. Fertil Steril (2017) 107:641–8.e2. doi: 10.1016/j.fertnstert.2016.12.005 28108009

[4] Venetis C Kolibianakis E Bosdou J Lainas G Sfontouris I Tarlatzis B. Estimating the net effect of progesterone elevation on the day of hCG on live birth rates after IVF: a cohort analysis of 3296 IVF cycles. Hum Reprod (Oxford England) (2015) 30:684–91. doi: 10.1093/humrep/deu362 25586787

[5] Fang T Su Z Wang L Yuan P Li R Ouyang N. Predictive value of age-specific FSH levels for IVF-ET outcome in women with normal ovarian function. Reprod Biol Endocrinol RB&E (2015) 13:63. doi: 10.1186/s12958-015-0056-6 26082101 PMC4470037

[6] van Loendersloot LL van Wely M Limpens J Bossuyt PM Repping S van der Veen F. Predictive factors in *in vitro* fertilization (IVF): a systematic review and meta-analysis. Hum Reprod Update (2010) 16:577–89. doi: 10.1093/humupd/dmq015 20581128

[7] Bosch E Ezcurra D. Individualised controlled ovarian stimulation (iCOS): maximising success rates for assisted reproductive technology patients. Reprod Biol Endocrinol (2011) 9:82. doi: 10.1186/1477-7827-9-82 21693025 PMC3150250

[8] Fatemi HM Blockeel C Devroey P. Ovarian stimulation: today and tomorrow. Curr Pharm Biotechnol (2012) 13:392–7. doi: 10.2174/138920112799362007 21657998

[9] Braga D Zanetti BF Setti AS Iaconelli A Jr. Borges E Jr. Immature oocyte incidence: Contributing factors and effects on mature sibling oocytes in intracytoplasmic sperm injection cycles. JBRA Assist Reprod (2020) 24:70–6. doi: 10.5935/1518-0557.20190056 PMC6993156 31589389

[10] Braga DP Figueira Rde C Ferreira RC Pasqualotto FF Iaconelli A Jr. Borges E Jr. Contribution of in-vitro maturation in ovarian stimulation cycles of poor-responder patients. Reprod Biomed Online (2010) 20:335–40. doi: 10.1016/j.rbmo.2009.12.009 20117048

[11] Shu Y Gebhardt J Watt J Lyon J Dasig D Behr B. Fertilization, embryo development, and clinical outcome of immature oocytes from stimulated intracytoplasmic sperm injection cycles. Fertil Steril (2007) 87:1022–7. doi: 10.1016/j.fertnstert.2006.08.110 17261289

[12] Wang M Yang Q Ren X Hu J Li Z Long R. Investigating the impact of asymptomatic or mild SARS-CoV-2 infection on female fertility and *in vitro* fertilization outcomes: A retrospective cohort study. EClinicalMedicine (2021) 38:101013. doi: 10.1016/j.eclinm.2021.101013 34250457 PMC8259363

[13] Gong J Ou J Qiu X Jie Y Chen Y Yuan L. A tool for early prediction of severe coronavirus disease 2019 (COVID-19): A multicenter study using the risk nomogram in wuhan and guangdong, China. Clin Infect Dis (2020) 71:833–40. doi: 10.1093/cid/ciaa443 PMC7184338 32296824

[14] Van Calster B Wynants L Verbeek JFM Verbakel JY Christodoulou E Vickers AJ. Reporting and interpreting decision curve analysis: A guide for investigators. Eur Urol (2018) 74:796–804. doi: 10.1016/j.eururo.2018.08.038 30241973 PMC6261531

[15] Tal R Tal O Seifer BJ Seifer DB. Antimüllerian hormone as predictor of implantation and clinical pregnancy after assisted conception: a systematic review and meta-analysis. Fertil Steril (2015) 103:119–30.e3. doi: 10.1016/j.fertnstert.2014.09.041 25450298

[16] Zhang Z Zhu LL Jiang HS Chen H Chen Y Dai YT. Predictors of pregnancy outcome for infertile couples attending IVF and ICSI programmes. Andrologia (2016) 48:874–81. doi: 10.1111/and.12525 26781087

[17] Meseguer M Herrero J Tejera A Hilligsøe KM Ramsing NB Remohí J. The use of morphokinetics as a predictor of embryo implantation. Hum Reprod (Oxford England) (2011) 26:2658–71. doi: 10.1093/humrep/der256 21828117

[18] Tandara M Bajić A Tandara L Bilić-Zulle L Šunj M Kozina V. Sperm DNA integrity testing: big halo is a good predictor of embryo quality and pregnancy after conventional IVF. Andrology (2014) 2:678–86. doi: 10.1111/j.2047-2927.2014.00234.x 24947544

[19] Yuan X Saravelos SH Wang Q Xu Y Li TC Zhou C. Endometrial thickness as a predictor of pregnancy outcomes in 10787 fresh IVF-ICSI cycles. Reprod Biomed Online (2016) 33:197–205. doi: 10.1016/j.rbmo.2016.05.002 27238372

[20] Tokgoz VY Tekin AB. Serum progesterone level above 0.85 ng/mL and progesterone/estradiol ratio may be useful predictors for replacing cleavage-stage with blastocyst-stage embryo transfer in fresh IVF/ICSI cycles without premature progesterone elevation. Arch Gynecol Obstet (2021) 305:1011–19. doi: 10.1007/s00404-021-06304-3 34716819

[21] Li J Qian WP Sun QY. Cyclins regulating oocyte meiotic cell cycle progression†. Biol Reprod (2019) 101:878–81. doi: 10.1093/biolre/ioz143 PMC6877757 31347666

[22] Masui Y. From oocyte maturation to the *in vitro* cell cycle: the history of discoveries of maturation-promoting factor (MPF) and cytostatic factor (CSF). Differ Res Biol Diversity (2001) 69:1–17. doi: 10.1046/j.1432-0436.2001.690101.x 11776390

[23] Adhikari D Liu K. The regulation of maturation promoting factor during prophase I arrest and meiotic entry in mammalian oocytes. Mol Cell Endocrinol (2014) 382:480–7. doi: 10.1016/j.mce.2013.07.027 23916417

[24] Ahmed TA Ahmed SM El-Gammal Z Shouman S Ahmed A Mansour R. Oocyte aging: The role of cellular and environmental factors and impact on female fertility. Adv Exp Med Biol (2020) 1247:109–23. doi: 10.1007/5584_2019_456 31802446

[25] Zhao H Li T Zhao Y Tan T Liu C Liu Y. Single-cell transcriptomics of human oocytes: Environment-driven metabolic competition and compensatory mechanisms during oocyte maturation. Antioxid Redox Signaling (2019) 30:542–59. doi: 10.1089/ars.2017.7151 PMC6338670 29486586

[26] Warzych E Lipinska P. Energy metabolism of follicular environment during oocyte growth and maturation. J Reprod Dev (2020) 66:1–7. doi: 10.1262/jrd.2019-102 31787727 PMC7040205

[27] Roth Z. Symposium review: Reduction in oocyte developmental competence by stress is associated with alterations in mitochondrial function. J Dairy Sci (2018) 101:3642–54. doi: 10.3168/jds.2017-13389 29395145

[28] Yuan P Guo Q Guo H Lian Y Zhai F Yan Z. The methylome of a human polar body reflects that of its sibling oocyte and its aberrance may indicate poor embryo development. Hum Reprod (Oxford England) (2021) 36:318–30. doi: 10.1093/humrep/deaa292 33313772

[29] Liu Z Xi Q Zhu L Yang X Jin L Wang J. TUBB8 mutations cause female infertility with Large polar body oocyte and fertilization failure. Reprod Sci (Thousand Oaks Calif) (2021) 28:2942–50. doi: 10.1007/s43032-021-00633-z 34160777

[30] Liu Z Zhu L Wang J Luo G Xi Q Zhou X. Novel homozygous mutations in PATL2 lead to female infertility with oocyte maturation arrest. J Assist Reprod Genet (2020) 37:841–7. doi: 10.1007/s10815-020-01698-6 PMC7183019 32048119

[31] Walls ML Hart RJ. In vitro maturation. Best Pract Res Clin Obstet Gynaecol (2018) 53:60–72. doi: 10.1016/j.bpobgyn.2018.06.004 30056110

[32] Son WY Henderson S Cohen Y Dahan M Buckett W. Immature oocyte for fertility preservation. Front Endocrinol (Lausanne) (2019) 10:464. doi: 10.3389/fendo.2019.00464 31379739 PMC6650526

[33] Black M Liu DY Bourne H Baker HW. Comparison of outcomes of conventional intracytoplasmic sperm injection and intracytoplasmic sperm injection using sperm bound to the zona pellucida of immature oocytes. Fertil Steril (2010) 93:672–4. doi: 10.1016/j.fertnstert.2009.08.063 19878934

[34] Izadi M Khalili M Salehi-Abargouei A Rezvani M Aflatoonian B. Use of zona pellucida-bound spermatozoa as a natural selection in improvement of ICSI outcomes: A systematic review and meta-analysis. Andrologia (2021) 53:e14022. doi: 10.1111/and.14022 33942906

[35] Sang Q Li B Kuang Y Wang X Zhang Z Chen B. Homozygous mutations in WEE2 cause fertilization failure and female infertility. Am J Hum Genet (2018) 102:649–57. doi: 10.1016/j.ajhg.2018.02.015 PMC5985286 29606300

[36] Wang M Zhu L Liu C He H Wang C Xing C. A novel assisted oocyte activation method improves fertilization in patients with recurrent fertilization failure. Front Cell Dev Biol (2021) 9:672081. doi: 10.3389/fcell.2021.672081 34368125 PMC8334862

[37] Lawrenz B Labarta E Fatemi H Bosch E. Premature progesterone elevation: targets and rescue strategies. Fertil Steril (2018) 109:577–82. doi: 10.1016/j.fertnstert.2018.02.128 29653703

[38] Wang M Xi Q Yang Q Li Z Yang L Zhu L. The relationship between a novel evaluation parameter of premature luteinization and IVF outcomes. Reprod Biomed Online (2021) 42:323–31. doi: 10.1016/j.rbmo.2020.10.009 33250412

[39] Borman SM Chaffin CL Schwinof KM Stouffer RL Zelinski-Wooten MB. Progesterone promotes oocyte maturation, but not ovulation, in nonhuman primate follicles without a gonadotropin surge. Biol Reprod (2004) 71:366–73. doi: 10.1095/biolreprod.103.023390 14985242

[40] Urrego R Herrera-Puerta E Chavarria NA Camargo O Wrenzycki C Rodriguez-Osorio N. Follicular progesterone concentrations and messenger RNA expression of MATER and OCT-4 in immature bovine oocytes as predictors of developmental competence. Theriogenology (2015) 83:1179–87. doi: 10.1016/j.theriogenology.2014.12.024 25662108

[41] Xi Q Yang Q Wang M Huang B Zhang B Li Z. Individualized embryo selection strategy developed by stacking machine learning model for better *in vitro* fertilization outcomes: an application study. Reprod Biol Endocrinol (2021) 19:53. doi: 10.1186/s12958-021-00734-z 33820565 PMC8020549

[42] Curchoe CL Bormann CL. Artificial intelligence and machine learning for human reproduction and embryology presented at ASRM and ESHRE 2018. J Assist Reprod Genet (2019) 36:591–600. doi: 10.1007/s10815-019-01408-x 30690654 PMC6504989

[43] Zhu L Li J Wang M Fang Z Zheng F Li Z. Normalized mitochondrial DNA copy number can optimize pregnancy outcome prediction in IVF. Reprod Sci (Thousand Oaks Calif) (2021) 28:1439–46. doi: 10.1007/s43032-020-00422-0 33400212

[44] Zhang B Cui YQ Wang M Li JJ Jin L Wu DR. *In vitro* fertilization (IVF) cumulative pregnancy rate prediction from basic patient characteristics. IEEE Access (2019) 7:130460–7. doi: 10.1109/ACCESS.2019.2940588

